# Theoretical and Electrochemical Evaluation of *Cannabis Sativa L*. Extracts as Corrosion Inhibitors for Mild Steel in Acidic Medium

**DOI:** 10.1002/open.202400273

**Published:** 2024-12-23

**Authors:** Salima Haddou, Kaoutar Zaidi, Omar Dagdag, Asmae Hbika, Mohamed Adil Mahraz, Mohamed Bouhrim, Ali S. Alqahtani, Omar M. Noman, Hansang Kim, Abdelouahad Aouniti, Belkheir Hammouti, Abdelkrim Chahine

**Affiliations:** ^1^ Laboratory of Advanced Materials and Process Engineering Faculty of Science University Ibn Tofail University Street Kenitra B.P 242 Morocco; ^2^ Laboratory of Applied Chemistry and Environment (LCAE) Faculty of Sciences University Mohammed 1st Oujda 60000 Morocco; ^3^ Department of Mechanical Engineering Gachon University Seongnam 13120 Republic of Korea; ^4^ Laboratory of Engineering, Electrochemistry, Modeling and Environment Faculty of Sciences Dhar El Mahraz Sidi Mohammed Ben Abdellah University Fez 30050 Morocco; ^5^ Laboratory of Biological Engineering, Team of Functional and Pathological Biology Faculty of Sciences and Techniques Beni Mellal University Sultan Moulay Slimane Beni Mellal Morocco; ^6^ Department of Pharmacognosy College of Pharmacy King Saud University Riyadh Saudi Arabia; ^7^ Euromed University of Fes, (UEMF) BP. 15 30000 Fez Morocco

**Keywords:** Cannabis *sativa* L, Corrosion inhibition, Mild steel, Acidic medium, Electrochemical techniques, Theoretical approach

## Abstract

The corrosion of metals in acidic environments remains a significant challenge, driving the search for sustainable and eco‐friendly inhibitors derived from natural sources. This study evaluates the corrosion inhibition potential of three extracts from Cannabis *sativa* L., namely ethanol extract (EET), hexane extract (EHX), and dichloromethane extract (EDM), for mild steel in a 1 M HCl acidic medium. The investigation employed weight loss (WL) measurements, electrochemical impedance spectroscopy (EIS), and potentiodynamic polarization (PDP) techniques. To understand their inhibitive performance, density functional theory (DFT) was used. For a more comprehensive theoretical analysis, Monte Carlo (MC) and molecular dynamics (MD) simulations were used. The corrosion inhibition efficiency increased with the increase of EET, EHX, and EDM concentrations up to 91 %, 89 %, and 83 %, respectively, obtained at 308 K for a 0.8 g/L concentration. Polarization studies classify EET, EHX, and EDM as mixed‐type inhibitors with a predominantly anodic effect, functioning through adsorption on the metal surface. The adsorption of these extracts on mild steel conforms to the Langmuir isotherm model, with adsorption equilibrium constants (K_ads_) of 3.0143 M, 5.1245 M, and 2.2009 M for EET, EHX, and EDM, respectively, highlighting their potential as effective corrosion inhibitors. The EET extract exhibits a high activation energy (E_a_) of 101.70 kJ/mol, while the EHX and EDM extracts show E_a_ values of 79.05 kJ/mol and 82.93 kJ/mol, respectively, all significantly higher than the E_a_ of blank, which is 30.23 kJ/mol, indicating that the extracts effectively inhibit corrosion by increasing the activation energy, with EET being the most potent inhibitor. Theoretical approaches based on DFT, MC, and MD simulations clearly explain the mode of adsorption of the majority of molecules on the metal surface. The inhibition process may result from a synergistic intermolecular effect of the major compounds in the extract, which interact at various active adsorption sites on the metal surface. Simulations indicate that catechin dihydrate in EET (52.42 %), linoleic acid in EHX (42.92 %), and naringenin in EDM (41.92 %) are close to the metal surface, suggesting strong interactions with the material. The results obtained from experimental measurements and theoretical calculations agree, highlighting the potential for developing more sustainable corrosion inhibitors based on plant‐derived compounds.

## Graphical abstract

This study examines ethanol (EET), hexane (EHX), and dichloromethane (EDM) extracts of *C. sativa L*. as eco‐friendly corrosion inhibitors for mild steel in 1 M HCl. At 0.8 g/L, inhibition efficiencies of 90 %, 88 %, and 81 % are observed. Adsorption follows the Langmuir isotherm, supported by DFT, Monte Carlo, and molecular dynamics simulations.

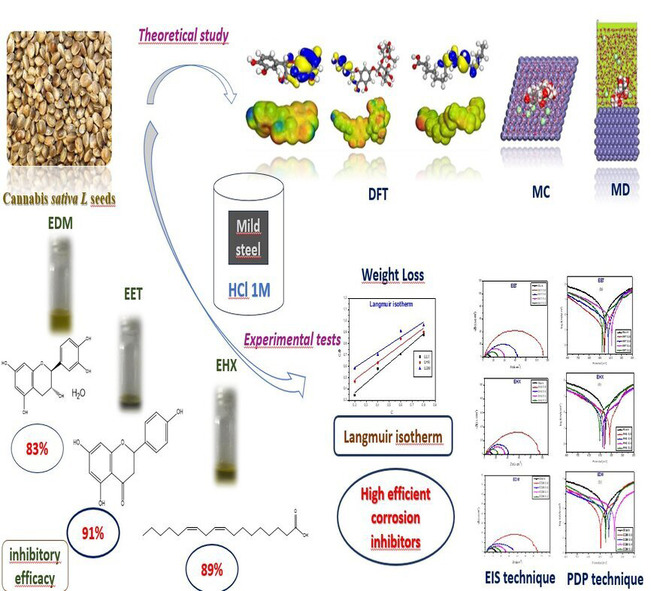

## 1 Introduction

Mild steel materials find applications across various industries including automobile, chemical, and related sectors, where they are used for handling acids, bases, and salt solutions.[^1^, ^2^] Their widespread adoption is attributed to their favorable mechanical properties and cost‐effectiveness.[^2^, ^3^] Therefore, Researchers have long been committed to studying the corrosion of mild steel in diverse corrosive environments.[^4^, ^5^] Mild steel possesses numerous properties that make it suitable for various industries including but not limited to chemical, electrochemical, food processing, petroleum, and power production.[^6^, ^7^, ^8^, ^9^, ^10^] However, degradation occurs due to severe corrosion attacks on mild steel surfaces that are stripped in aqueous acidic conditions. Hydrochloric acid (HCl) is frequently employed as a solvent for chemical cleaning, affecting a wide range of scales.[^10^, ^11^] Within HCl solutions, inhibitors primarily consist of nitrogen compounds.^[12]^ The effectiveness of organic compounds as a corrosion inhibitor is firmly established to depend not only on the characteristics of the operating environment, the condition of the electrochemical potential at the interface, and the metal surface but also on the structural attributes of the inhibitor. Organic molecules utilized as corrosion inhibitors typically comprise heteroatoms such as oxygen, nitrogen, and sulfur. These heteroatoms, owing to their lone pair electrons, can occupy the vacant d orbitals of the metal surface when immersed in an aggressive medium.[^13^, ^14^] However, most of these inhibitors are synthetic organic compounds, which are expensive, environmentally toxic, and pose negative health impacts, despite their significant inhibitive properties.[^15^, ^16^, ^17^] In recent years, corrosion researchers have increasingly turned to plant extracts as ecological corrosion inhibitors (green inhibitors’) as a substitute for synthetic ones, aiming to address environmental concerns. In recent years, the medical use of cannabis has become more widespread. Cannabis *sativa L* has antihypertensive, antioxidant, and hypotensive effects.^[18]^ It is also used to treat glaucoma, multiple sclerosis, and chemotherapy. Extracts of the plant are also used to prevent corrosion. At present, the cannabis plant is considered an industrial crop in Morocco. This is why its cultivation is supported and encouraged at the national level.^[19]^ The high nutritional content of hemp seed makes it one of the most varied food sources in terms of nutrients. It can be eaten as is (whole seed, hulled), as well as processed products such as oil, flour, and protein powder.^[20]^ Cannabis seed oil is a natural product of great interest as a raw material for various industries such as pharmaceuticals, cosmetics, perfumery, and food processing.^[21]^ Around 35 % of the oil is present in cannabis seeds, which are renowned for their richness in polyunsaturated fatty acids (over 85 %). Cannabis seeds offer a large amount of storage proteins and high arginine and glutamic acid levels. and have also been reported in seeds with high radical scavenging properties.^[22]^ Cannabis seed oil has a variety of industrial applications, including printing inks, wood preservatives, detergents, and soaps.^[23]^ Cannabis, renowned for its therapeutic benefits, is made up of multiple components, each with unique effects. Flowers, leaves, roots and stems encompass a range of cannabinoids and terpenes, each associated with specific medicinal uses. While many studies have examined these effects, recent studies have documented a series of beneficial effects associated with cannabis. The flowers, rich in cannabinoids such as THC and CBD, are particularly well known for their ability to relieve chronic pain and reduce anxiety, due to their psychotropic and anxiolytic characteristics.[^24^, ^25^] At the same time, the leaves of the cannabis plant possess antioxidant and anti‐inflammatory properties, contributing to the reduction of oxidative stress and inflammation, proving useful in the management of diseases such as arthritis.[^26^, ^27^] Although less often mentioned, the roots also have medicinal benefits, offering both analgesic and anti‐inflammatory effects.^[28]^ Finally, cannabis resin, rich in concentrated cannabinoids, offers powerful therapeutic effects, notably in treating epilepsy and severe pain.^[29]^ So, while the flowers are often considered the most beneficial part due to their wealth of active compounds.^[30]^ Extracts of the plant are also used to prevent corrosion.^[31]^ However, for food purposes, this is due to the high concentration of polyunsaturated fatty acids in the seeds that makes these products particularly intriguing.^[21]^ The studies that have been carried out suggest that cannabis extract is a powerful inhibitor of nickel corrosion in a sulfuric acid environment. Cannabis *sativa L* extracts play a highly effective role as a cathodic inhibitor of copper corrosion in aerated 0.5 M H_2_SO_4_, and studies in this field confirm that inhibition increases as the concentration of cannabis extract increases.^[32]^ Cannabis *sativa L* seeds have a high fat content, as pointed out by.^[33]^ Extracts of Cannabis *sativa* have recently attracted growing interest as corrosion inhibitors for mild steel in acidic environments, particularly hydrochloric acid (HCl). Several studies have evaluated the inhibitory efficacy of different parts of the plant, including leaves, flowers, and seeds. For example, a study by Zhang et al., showed that Cannabis *sativa* leaf extracts, obtained by ethanol extraction, had a good capacity to inhibit corrosion in a 1 M HCl solution, with an efficacy of up to 72 %.^[34]^ These results were corroborated by electrochemical tests, including potentiodynamic polarization and electrochemical impedance (EIS), which showed a significant reduction in the rate of corrosion. Another study by Zheng et al., evaluated the corrosion inhibition of Cannabis *sativa* seed and flower extracts in an acidic environment. They found that flowers, in particular, showed a more marked inhibitory activity than seeds, with an efficacy of up to 80 %.^[35]^ The tests used weight loss methods and electrochemical analysis to quantify inhibitory efficacy. In contrast, Haldhar et al., examined the influence of Cannabis *sativa* roots in an HCl solution and observed a moderate inhibitory efficacy of around 60 %,^[36]^ suggesting that the roots may be less active than other parts of the plant for this specific application. The inhibitory mechanisms appear to be linked to the presence of phenolic compounds and alkaloids, which interact with metal surfaces, thereby reducing the rate of corrosion Damej et al.,.^[37]^ These studies highlight not only the effectiveness of Cannabis *sativa* extracts but also the variability of their inhibitory potential depending on the parts of the plant used.

The comparative table (Table 1) presents the main differences and similarities between our study and those of other researchers concerning the use of Cannabis *sativa* extracts as corrosion inhibitors for mild steel in acidic environments. Our study stands out for the integration of theoretical (DFT modeling) and electrochemical (potentiodynamic polarization, EIS) approaches, offering a complete analysis of the inhibitory mechanisms, with efficiencies ranging from 81 to 90 %. In contrast, other studies, such as those by Zhang et al., focus mainly on electrochemical experimental tests, with inhibitory efficiencies ranging from 55 to 70 %. Damej et al., and Zheng et al., also combine theory and experimentation, obtaining similar or slightly better results, up to 80–85 %. What particularly distinguishes our work is the more detailed approach and the in‐depth molecular modeling, allowing a better understanding of the corrosion mechanisms and the effectiveness of the extracts.

**Table 1:** Comparative table of studies on Cannabis *sativa* as a corrosion inhibitor for mild steel in acid environments.

Approach used	Extraction method	Part of the plant	Corrosive environment	Experimental techniques	Inhibitory efficacy (%)	References
Theoretical and electrochemical	Maceration technique	Cannabis seed	Hydrochloric acid (HCl)	Electrochemical impedance spectroscopy (EIS) and potentiodynamic polarization (PDP)	81 %–90 %	our study
Electrochemical, without theoretical approach	Extraction with ethanol	Leaves	Sulphuric acid (H₂SO₄)	Potentiodynamic polarization, Weight loss trial	60–70 %	[34]
Theoretical and electrochemical	Aqueous maceration	Leaves, Flowers	Hydrochloric acid (HCl)	Potentiodynamic polarization, Electrochemical impedance spectroscopy (EIS)	65–75 %	[37]
Theoretical and electrochemical	Ethanol extraction	Sheets	Sulphuric acid (H₂SO₄)	Potentiodynamic polarization, Electrochemical impedance spectroscopy (EIS)	80–85 %	[35,38]

The aim was to develop this crop as a non‐conventional oilseed, offering new sources of oils that could be exploited in the food and industrial sectors. Also, is to investigate the inhibitory effects of Cannabis *sativa L* ethanol extract (EET), Cannabis *sativa L* hexane extract (EHX), and Cannabis *sativa L* dichloromethane extract (EDM) on the corrosion of mild steel in a corrosive solution of 1 M HCl. Various techniques were employed for this investigation. Weight loss measurements were utilized to identify the adsorption isotherm. Electrochemical Impedance Spectroscopy (EIS) was used to investigate the corrosion process of mild steel both before and after the addition of EET, EHX, and EDM inhibitors to the test solution, as well as to ascertain the polarization resistance of the mild steel specimens. Polarization curves (PDP) were registered to determine the corrosion current density and other electrochemical parameters, as well as thermodynamic activation parameters. Additionally, Density Functional Theory (DFT) and Molecular dynamics (MD) simulations were employed to give a more detailed theoretical analysis of inhibitory performance.

## Materials and Methods

### Extracts Preparation

Cannabis seeds were collected in the northern Moroccan town of Ketama in December 2021, the drying process generally takes about a week at room temperature (25 °C). To prepare cannabis seed extracts, Once the seeds have been isolated, they are carefully sieved to remove any impurities or debris, before being finely ground into powder using a grinder. Different extracts were obtained from seed powders by maceration according to the following extraction protocol: 65 g of crushed seeds were subjected to solid‐liquid extraction at room temperature and under magnetic stirring in the presence of 300 mL of hexane according to the method developed by Salima et al.^[39]^ Filter the solution under vacuum with a Buchner funnel, recover the filtrate, and then filter it with a filter crucible to obtain a good filtrate. The filtrate passed through a steam rotor to evaporate the solvent and recover the oil separately. After evaporation and drying of the filtrate in an oven set at 35 °C, the dichloromethane extract, the ethanolic extract and the aqueous extract were prepared using the same method, the only variation being the maceration time. After preparation, the extracts were stored in opaque bottles at −4 °C until ready for use.^[39]^

### Electrolyte and Mild Steel Specimens

The material under investigation was a mild steel (MS) coupon with the following composition (% by mass): 0.21 % carbon (C), 0.38 % silicon (Si), 0.05 % manganese (Mn), 0.09 % phosphorus (P), 0.05 % sulfur (S), 0.01 % aluminum (Al), and the remainder iron (Fe). These coupons were utilized for both electrochemical tests and weight loss studies. For the weight loss study, MS sheets were cut into pieces measuring 1.5×1.5×0.3 cm, while for the electrochemical study, mild steel electrodes were prepared with an exposed area of 0.5 cm^2^ to the acid solution. The MS underwent polishing using abrasive paper ranging from mesh sizes 120 to 2000, followed by sequential washing with distilled water and acetone to eliminate any organic residues, and then dried using hot air. The corrosive solution used for testing was a commercially available hydrochloric acid (HCl) solution (37 %). This acid was diluted with distilled water to a concentration of 1 M, to prepare the inhibitor concentrations tested, the appropriate quantity of each of the three C. *sativa* L extracts was weighed using the electronic balance and then dissolved in the appropriate volume of the blank solution, and these solutions were stirred until complete solubilization to obtain homogeneous solutions of concentrations of 0.8, 0.6, 0.4 and 0.2 g/L, respectively.

### Methods and Techniques

#### Weight Loss (WL) Method

Gravimetry is a foundational and extensively employed method in the realm of corrosion research to measure the alterations in mass undergone by a metallic material as a result of corrosion.^[40]^ This method relies on the precise measurement of the weight loss or gain of a metal sample exposed to a corrosive environment over a specified period, but it does not allow for the understanding of the underlying mechanisms or instantaneous parameters involved in corrosion. In this method, the decrease in mass of the MS coupons following corrosion and inhibition procedures at different concentrations of the extracts of C. *sativa L* was quantified, and these measurements were utilized to determine several key parameters including corrosion rate, corrosion efficiency, and adsorption degree at a specific temperature. The immersion duration was set at 6 hours, with a temperature of 308 K. The corrosion rate (***W***_***corr***_) (mg/h ⋅ cm^2^), the inhibitory efficiency (***IE***_***w***_), and surface coverage (**θ**) were computed using the following equations.[^41^, ^42^, ^43^, ^44^]

(1) $$W_{corr}=\frac{m_{i}-m_{f}}{S\times t}$$

(2) $${IE}_{w}=\frac{W_{corr}-W_{corr/inh}}{W_{corr}}\times 100$$

(3) $$\theta =1-\frac{W_{corr/inh}}{W_{corr}}$$

#### Potentiodynamic Polarization and Electrochemical Impedance Spectroscopy Techniques

To delve into the reaction mechanism involved in corrosion processes, both stationary and transient electrochemical tests were conducted. The electrochemical cell utilized for these tests was a PGZ100 potentiostat/galvanostat, which was monitored by a computer via Volta Master 4 software. This setup included three standard Pyrex® glass electrodes: a saturated calomel electrode (SCE) serving as the reference electrode, and a platinum head acting as the counter electrode with a surface area of 1.0 cm^2^. The working electrode (WE) consisted of mild steel (MS) in a cylindrical configuration with a lateral surface area of 0.5 cm^2^. The open circuit potential (OCP) was monitored for up to 30 minutes. Consequently, the steady‐state OCP denoted as E_OCP_, was determined before each test. During all electrochemical tests, potential‐current curves were recorded from the cathodic to the anodic direction within a range of −200 mV to −800 mV/SCE, employing a scan rate of 0.5 mV/s in both inhibited and uninhibited solutions. Electrochemical impedance spectroscopy (EIS) experiments were carried out over a frequency range of 100 kHz to 10 mHz using AC signals with an amplitude of 10 mV. These EIS experiments were performed at the open circuit potential (OCP). The efficiency of inhibition provided by the inhibitor was determined by calculating the values of charge transfer resistance, employing the equation 4.
(4)

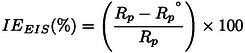




Where *R_p_* and 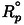 are polarization resistance in the presence and absence of inhibitor respectively.

The level of corrosion protection provided by the chosen extract's inhibitor was evaluated utilizing the subsequent expression:

(5) $${IE}_{PDP}\left(\%\right)=\left(\frac{{\begin{array}{c} i \end{array}}_{corr}^{\circ }{-i}_{corr}}{{\begin{array}{c} i \end{array}}_{corr}^{\circ }}\right)\times 100$$

Where *i_corr_* ^*°*^ and *i_corr_* represent, respectively, the current densities before and after the addition of the inhibitor.

#### Quantum Calculation Method

DFT estimations were performed in Biovia Materials Studio with the Dmol_3_ module.[^45^, ^46^] Generalized gradient approximation^[46]^ employing M11‐L[^47^, ^48^] and DND 3.5/COSMO (water) model)^[49]^ was used for geometry optimizations.

The examination and understanding of interactions between the EET, EHX, and EDM molecules and the MS surface were conducted through Monte Carlo (MC) and Molecular Dynamics (MD) simulations. For the Monte Carlo (MC) and Molecular Dynamics (MD) simulations, a periodic boundary condition (PBC) slab of Fe[0,1,1] was constructed with dimensions of 24.823×24.823×45 Å, including a 30 Å vacuum layer. The system was packed using MC simulations with an inhibitor molecule, 750 water molecules, 5 chloride ions, and 5 hydronium ions. Both MD and MC simulations were conducted using the Condensed Phase Optimized Molecular Potential III (COMPASSIII) force field.[^50^, ^51^, ^52^] Molecular Dynamics (MD) simulations were carried out using the canonical NVT ensemble (constant volume, constant temperature) at 298 K, with a total simulation time of 1500 ps and a time step of 1 fs. Temperature regulation was maintained throughout the simulation using the Berendsen thermostat, ensuring stable thermal conditions. These simulations are commonly employed to explore various molecular interactions occurring between the estragol, linalol, and methyleugenol molecules and the MS surface. Materials Studio 8.0 software was used to perform the MC and MD simulations.[^50^, ^51^, ^52^]

## 2 Results and Discussion

**Figure 1:**
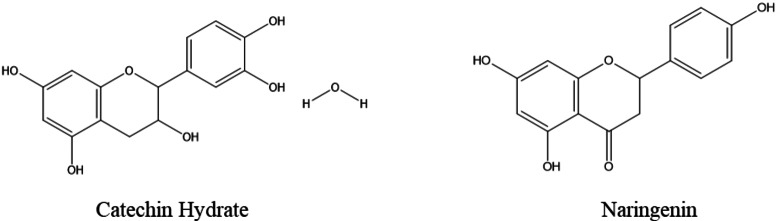
Chemical structure of naringenin, the majority compound in EET, and catechin hydrate in EDM.

In a previous study, the EDM and EET extracts were analyzed using high‐performance liquid chromatography,[^53^, ^54^] while EHX was examined via gas chromatography.^[39]^ The dichloromethane extract was found to be predominantly rich in catechin dihydrate, constituting approximately 52.42 % of its composition. Meanwhile, the primary component in the ethanolic extract was identified as naringenin, comprising 41.92 % of the extract, followed by Rutin at 10.08 % (Figure 1).

**Figure 2:**
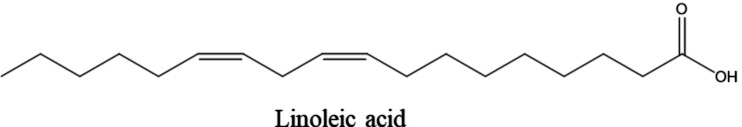
Chemical structure of linoleic acid, the main fatty acid in EHX.

Previously, a qualitative examination was conducted on the hexanic extract of Moroccan Cannabis seeds using GC‐MS gas chromatography,^[39]^ revealed the presence of six compounds, among these, linoleic acid was the most abundant at 42.92 %, followed by 7‐octadecenoic acid at 22.91 %, and palmitic acid at 15.37 % (Figure 2). Additionally, smaller quantities of linolenic acid, stearic acid, and heptacosanoic acid were also detected.

### 2.1 Gravimetric Tests

#### 2.1.1 Concentration Influence

The gravimetric method serves as the primary approach for assessing the inhibitive impact of a green corrosion inhibitor in retarding the corrosion process. The diverse outcomes acquired from the weight loss assessments, indicating corrosion rate, inhibitory efficiency and surface coverage across various concentrations at 308 K, are consolidated in Table 2.

**Table 2:** Corrosion rates and inhibition efficiency of mild steel in 1 M HCl before and after the addition of different concentrations of three major components of C. *sativa L* extract at 308 K.

Code	*Conc*. (g/L)	*W_corr_* (mg/cm^2^ h)	*IE_w_* (%)	*θ*
Blank	1 M	0.8024	–	–
EET	0.2	0.3372	58	0.58
	0.4	0.2482	69	0.69
	0.6	0.1231	85	0.85
	0.8	**0.0720**	**91**	**0.91**
EHX	0.2	0.4584	43	0.43
	0.4	0.2941	63	0.63
	0.6	0.2311	71	0.71
	0.8	**0.0900**	**89**	**0.89**
EDM	0.2	0.5270	34	0.34
	0.4	0.3470	57	0.57
	0.6	0.2744	66	0.66
	0.8	**0.1350**	**83**	**0.83**

The data obtained indicate a considerable reduction in the rate of corrosion of the three main components of C. *sativa L* extract. Conversely, the inhibition efficiency shows an increase according to increasing quantities of inhibitor concentration. The study revealed that the components EET, EHX, and EDM provided maximum protection of 91 %, 89 %, and 83 % at 0.8 g/L, respectively. From the obtained inhibitor efficiencies, we can conclude that the three components of C. *sativa L* extract exhibit good inhibition for MS, with a high value of *IE_W_* (%) for EET. This phenomenon can be elucidated by the adsorption of the three major components extracted from C. *sativa L* onto the surface of MS, thereby creating an insulating layer that shields it from the aqueous phase. This could be related to the formation of a protective film on the exposed area via the substitution of H_2_O molecules.[^55^, ^56^] For EET, the major component is Naringenin, which offers the highest efficiency (91 %) due to its optimal combination of an aromatic structure and multiple hydroxyl groups. These allow for strong adsorption on the metal surface, forming a stable and compact protective barrier. In the case of EHX, the major component is Linoleic acid, which provides a slightly lower efficiency (89 %) as its corrosion inhibition mechanism relies more on the formation of a hydrophobic barrier rather than strong electronic interactions with the metal. Finally, the major component of EDM is Catechin hydrate, which, while also an effective inhibitor (83 %), has a more complex structure that may not form as dense or uniform a protective layer as Naringenin, slightly reducing its overall effectiveness.

#### 2.1.2 Temperature Influence and Activation Parameters

Tests were conducted to determine kinetic parameters at three different temperatures (313 K, 323 K, and 333 K) in 1 M HCl solution by immersing mild steel samples for one hour at an optimal concentration of 0.8 g/L. Additionally, a blank solution experiment was conducted for comparison. The results from the Potentiodynamic Polarization (PDP) plots (Table 3) indicate that as temperature (T) rose, there was a corresponding increase in i_corr_, both with and without the presence of EET, EHX and EDM. This can be ascribed to the temperature‐induced decrease in the molecule's protective capability and alteration of the adsorption‐desorption equilibrium in favor of desorption. For instance, research on corrosion inhibitors such as fruit extracts and ginger extract at optimal concentrations shows that inhibition efficiency decreases with increasing temperature. This suggests that while these inhibitors are highly effective at lower temperatures, their efficiency diminishes as temperature rises. A similar behavior might be expected in the case of our C. *sativa L* extracts (EET, EDM, EHX). Comparing this temperature‐dependent behavior could provide valuable insights into the stability and adsorption strength of these inhibitors on the metal surface under different thermal conditions.[^57^, ^58^] Consequently, this shift diminishes the inhibitory effectiveness of these inhibitors. The kinetic parameters were computed utilizing the Arrhenius‐type plot and the transition state equation:[^59^, ^60^]

(6) $$i_{corr}=A\times exp\left(\frac{{-E}_{ads}}{RT}\right)$$

(7) $$i_{corr}=\frac{RT}{Nh}exp\left(\frac{{\Delta S}_{ads}}{R}\right)exp\left(\frac{{-\Delta H}_{ads}}{RT}\right)$$

**Table 3:** Electrochemical and thermodynamic characteristics were derived from PDP plots at various T (K) in both inhibitor‐free and inhibitor‐treated solutions (10^−3^ M).

Inh	*T* (K)	*E_corr_* (mv/SCE)	*I_corr_* (mA/cm^2^)	*IE_PDP_* (%)	*E_a_* (KJ/mol)	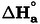 (KJ/mol)	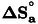 (J/mol. K)	*E_a_‐* 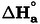
Blank	313	−480.2	1.4712	–	30.23	27.54	−153.65	2.7
	323	−480.2	2.4830	–				
	333	−428.1	2.9456	–				
EET	313	−434.8	0.1172	92	101.70	99.02	53.91	2.7
	323	−420.9	0.5080	77				
	333	−445.2	1.2190	59				
EHX	313	−445.2	0.2351	84	79.05	76.37	−12.74	2.7
	323	−408.1	0.7513	69				
	333	−376.5	1.4510	50				
EDM	313	−431.6	0.247	83	82.93	80.24	1.08	2.7
	323	−408.1	1.2108	51				
	333	−469.1	1.6538	44				

**Figure 3:**
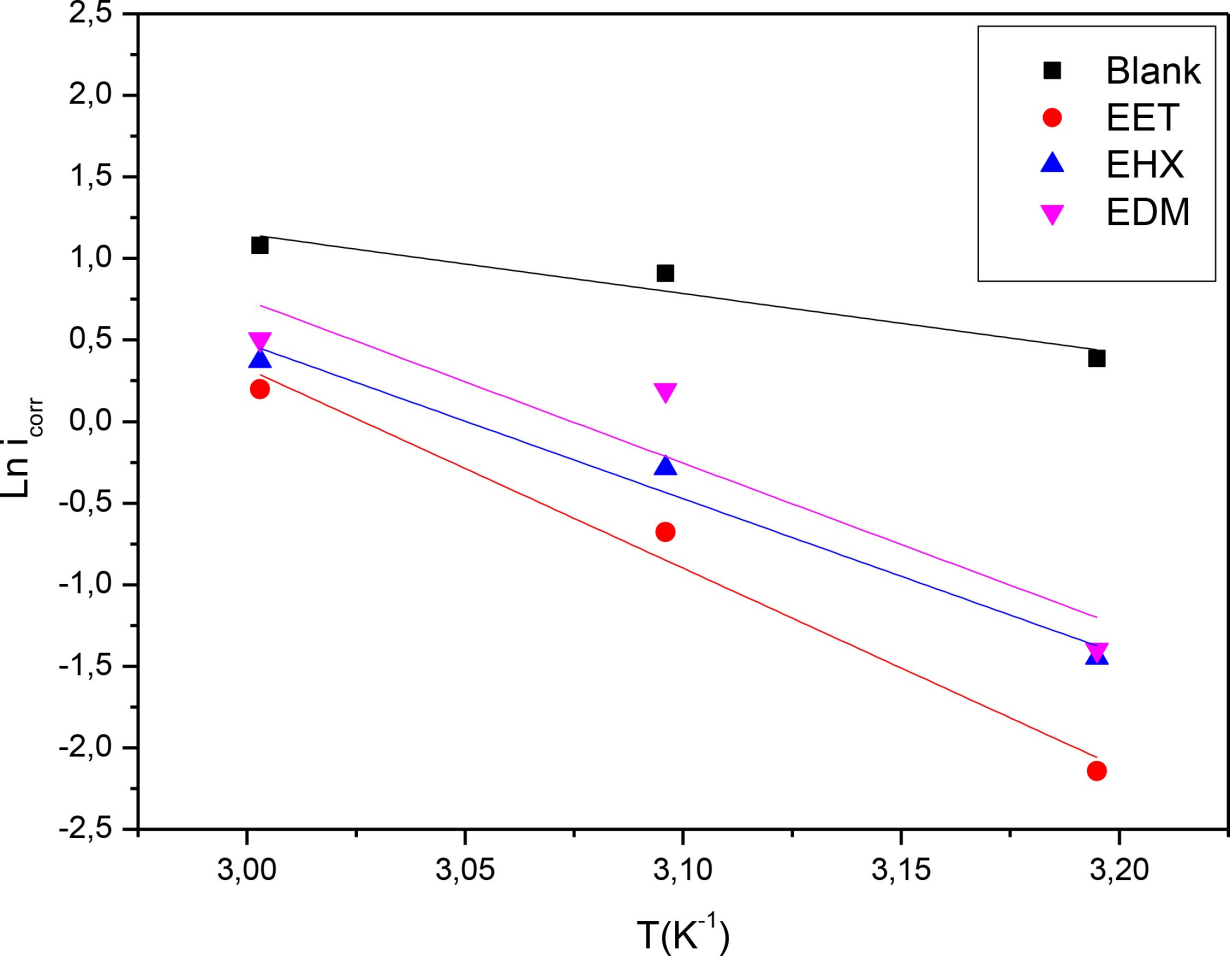
Arrhenius plot *ln i_corr_* versus 1/*T* at an optimum concentration of EET, EHX and EDM respectively.

**Figure 4:**
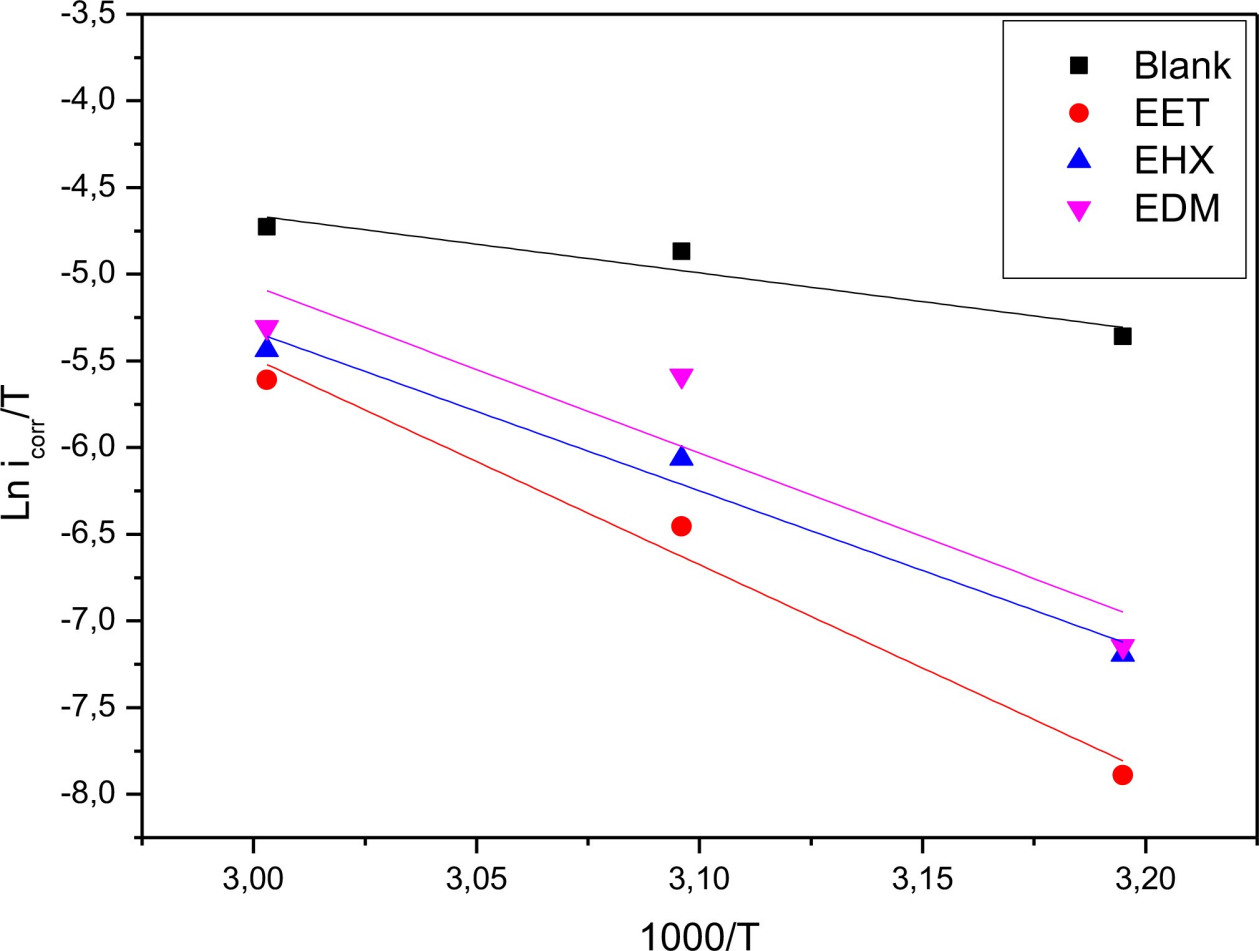
Arrhenius plot *ln i_corr_/T* versus 1000/T at optimum concentrations of EET, EHX, and EDM respectively.

The intercept and slope of straight lines of the plot of ln i_corr_ versus 1/*T* (Figure 3) can be used to calculate the Arrhenius factor and activation energy, similarly, a plot of *ln(i_corr_/T)* versus f(1000/*T*) allows for the determination of 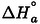 and 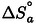 (Figure 4) as well which are tabulated in Table 4.

**Table 4:** EIS parameters were obtained from EIS curves of MS at various concentrations of EET, EHX, and EDM at 308 K.

Inh	Concentration (g.l^−1^)	*R_p_* (Ω.cm^2^)	*R_S_* (Ω.cm^2^)	*C_dl_* (μF/cm^2^)	*Q* (mF.S^n−1^)	*χ* ^*2*^ (10^−3^)	n	*IE_EIS_* (%)
	Blank	11.39	2.09	175.9	0.480	0.727	0.738	
EET	0.2	25.20	3.68	795.5	1.544	2.763	0.661	55
	0.4	36.95	3.32	254.0	0.485	6.690	0.755	69
	0.6	62.65	3.21	86.12	0.181	9.275	0.668	81
	0.8	111.1	2.81	57.26	0.118	8.325	0.848	**90**
EHX	0.2	21.91	2.37	264.3	2.732	1.836	0.655	48
	0.4	32.35	4.94	181.5	2.127	3.847	0.618	64
	0.6	38.73	4.29	122.9	1.403	5.806	0.719	71
	0.8	95.13	2.63	102.7	0.367	6.111	0.688	**88**
EDM	0.2	17.13	2.10	185.7	1.785	1.439	0.736	34
	0.4	24.02	2.27	146.5	0.509	2.615	0.634	53
	0.6	34.32	1.46	132.4	0.405	9.213	0.704	67
	0.8	63.87	2.57	124.5	0.316	9.285	0.604	**81**

The table indicates that the activation energy (*E_a_*) in the inhibitor‐containing solution surpasses that of the 1 M HCl solution, suggesting a reduction in mild steel dissolution due to the three major components of C. *sativa* L extract's formation of a protective barrier through adsorption on the metal surface.^[61]^ The positive enthalpy values 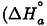 *)* signify the endothermic nature of the steel dissolution process.^[62]^ Moreover, the positive values of enthalpy 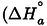 *)* in our results suggest that the steel dissolution process is endothermic, which is consistent with studies on other plant‐based inhibitors such as Moringa Oleifera Leaves Extract. These studies similarly report positive 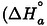 *)* values, attributing the corrosion inhibition to the strong adsorption of the plant extract components on the metal surface, which requires additional energy for the corrosion process to proceed.^[63]^

Furthermore, the neative 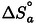 values for all three major components of C. *sativa* L extract indicate a propensity towards positive values, suggesting the overcoming of barriers to inhibitor adsorption on the mild steel surface.^[64]^ This behavior has also been reported for inhibitors such as Garlic extract, where the adsorption process is accompanied by a reduction in randomness due to the orderly arrangement of inhibitor molecules on the metal surface.^[65]^ Notably, the activation energy (*E_a_*) and enthalpy (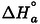 ) exhibit similar variations, affirming the thermodynamic relationship 8:^[66]^

(8)

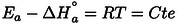




This finding corroborated the known thermodynamic of the monoatomic gas. The mechanism already proposed by Volmer step in the reduction of hydrogen ion ($H_{aq}^{+}$ ) to Hydrogen atom adsorbed ($H_{ads}$ ) at the metal surface:

(9) $$H_{aq}^{+}+e^{-}\to H_{ads}$$

### 2.2 Electrochemical Impedance Spectroscopy Technique

**Figure 5:**
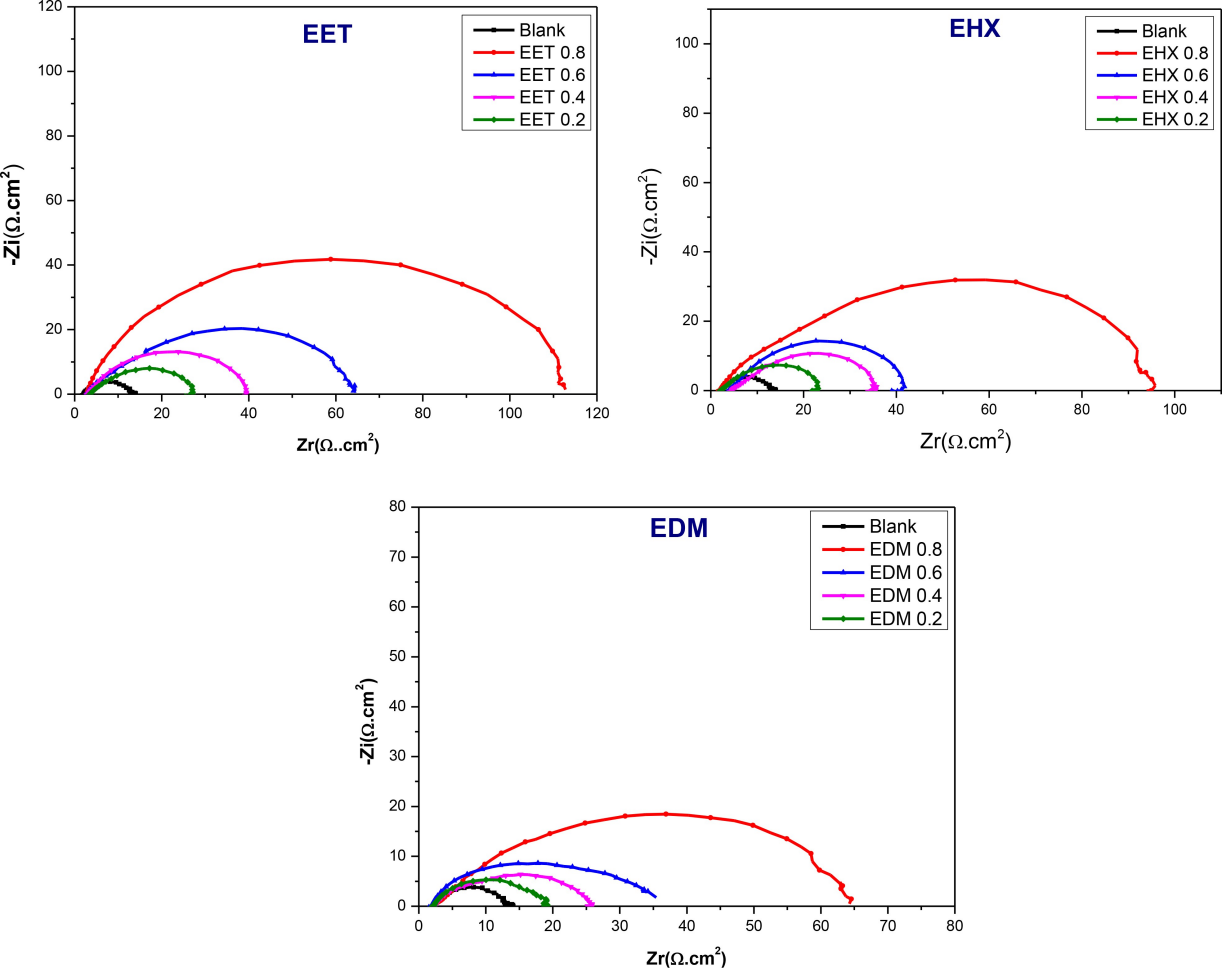
Nyquist plots for MS in acidic media with different concentrations of EET, EHX, and EDM inhibitors.

**Figure 6:**
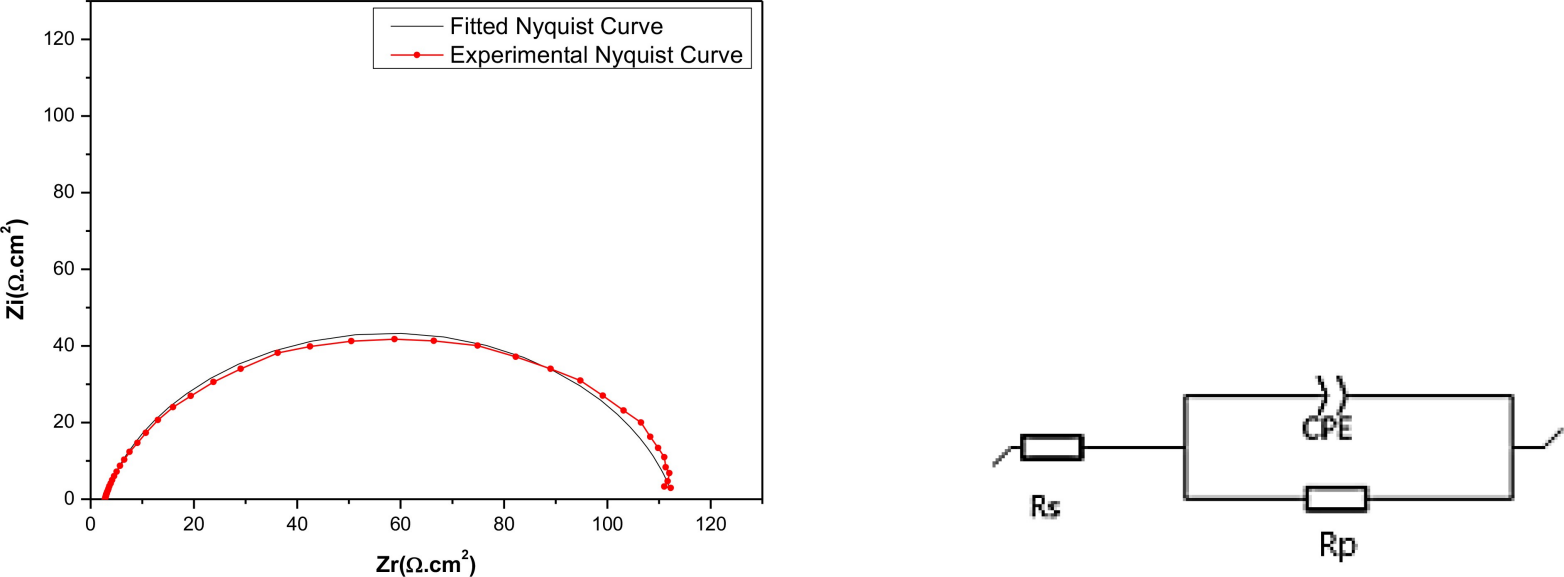
Equivalent electrical circuit and experimental and adjusted Nyquist representation of the mild steel/EET (0.8 g/L) interface in 1 M HCl.

To gain further insights into the kinetics of electrochemical processes occurring at the interface of mild steel and acid, particularly how these processes are altered in the presence of inhibitors, electrochemical impedance spectroscopy was utilized. Figure 5 exhibits Nyquist diagrams depicting the behavior of mild steel in an HCl solution, both with and without the presence of the three major components extracted from C. *sativa L*, after a 30 minutes exposure period at the open circuit potential (OCP). The Nyquist diagrams reveal a capacitive loop for various concentrations of EET, EHX, and EDM, indicating the presence of a single time constant and an imperfect semicircle at high frequencies. This suggests that the specimens studied in 1 M HCl exhibit capacitive behavior and are governed by the process of charge transfer.^[67]^ Additionally, the diagrams imply a porous and heterogeneous surface of mild steel, with the adsorption of inhibitors leading to frequency dispersion.^[68]^ The EIS parameters are organized in Table 4. Rp represents the diameter of the semi‐circles. As can be seen in Figure 5, the augmentation of the concentrations of the three major components of C. *sativa L* extract has increased the diameters of the semi‐circles which means increasing the *R_p_* values as it is presented in Table 4. Consequently, the inhibition efficiency (*IE_EIS_* (%)) was determined from the polarization resistance values in the tested solution, both in the absence (*R_p_°*) and presence (*R_p_*) of EET, EHX, and EDM (Eq. (4)). The equivalent electrical circuit (Figure 6) employed in the fitting comprises the resistance of the test solution (*R_s_*) in series with a Constant Phase Element (*CPE*), which is in parallel with the polarization resistance (*R_p_*) where (*R_p_*) includes the charge transfer resistance (*R_ct_*), diffuse layer resistance (*R_d_*) and accumulation resistance at the steel/solution interface (*R_a_*) which also provides a barrier effect, the total resistance is called as polarization resistance, *R_p_* (*R_p_=R_ct_+R_d_+R_a_*).^[69]^ This equivalent electrical circuit is justified by the low χ^2^ values obtained (in theory, the smaller *χ* ^*2*^ value is, the experimental data agreement is confirmed). Regarding the other parameters, an Ec‐lab software fitting of the experimental data was conducted to acquire the data presented in Table 4. In this context, *R_s_* represents the solution resistance, *Q* denotes the constant value of the constant phase element (*CPE*), and n corresponds to a phase shift, typically associated with surface inhomogeneity and deviation from an ideal capacitor.^[70]^ The other components have been described earlier. In the equivalent electrical circuit proposed for modeling the MS/solution interface, the *CPE* was substituted with a double layer capacitance (*C_dl_*) for a more accurate fit, as the *CPE* more effectively characterizes the real system compared to the *C_dl_* element, helping to reduce deviations caused by frequency dispersion.^[71]^ Nonetheless, the double‐layer capacitances (*C_dl_*) for a circuit incorporating a *CPE* were determined using the following formula:^[17]^

(10) $$C_{dl}{=(Q\times R_{p}^{1-n})}^{1/n}$$

The numerical value of n holds significance in elucidating the components of the circuit defined according to the *CPE*. Therefore, for exponent values of *n* equal to −1, 0, 0.5, or 1, this corresponds to an inductance, a resistance, Warburg impedance, or a capacitance, respectively. Incorporating the tested inhibitors into the acidic electrolyte results in a progressive reduction in the constant *Q* value of the *CPE*, subsequently diminishing the double‐layer capacitance (*C_dl_*). The observed decrease in the capacity of the double layer (*C_dl_*) upon the introduction of the investigated molecules signifies an expansion of the double layer's thickness and a reduction in the active metal surface.^[71]^ Additionally, *n* represents surface inhomogeneity, the values of n ranging between 0.604 and 0.848 indicate an increase in heterogeneity, reflecting the adsorption of ligands at the electrode‐electrolyte interface.[^72^, ^73^] In an ideal capacitor, n would be equal to 1. Upon analyzing the data presented in Table 4 and Figure 5, the results revealed that the addition of the three major components extracted from C. *sativa L* to the corrosive medium led to a significant increase in *R_p_*. This suggests that the inhibitors have a noticeable impact on reducing the corrosion rate of the steel in HCl solution, either by forming a protective barrier at the MS/HCl interface or by influencing the anodic dissolution reaction. Both *R_p_* and *IE_EIS_* (%) values increase proportionally with rising extract concentrations, up to 0.8 g/L in the acidic medium. This behavior is characteristic of organic molecules adsorbing onto the metal surface, indicating that the protective barrier becomes more effective as more extract molecules are present and adsorb onto the metal surface at higher concentrations. However, beyond 0.8 g/L, the *R_p_* and *IE_EIS_* (%) values decline, likely due to the formation of unstable surface films or soluble/unstable metal‐organic complexes. Among the tested concentrations, 0.8 g/L was optimal for EET, EHX, and EDM in the acidic solution, providing maximum protection efficiencies of 90 %, 88 %, and 81 %, respectively.

**Figure 7:**
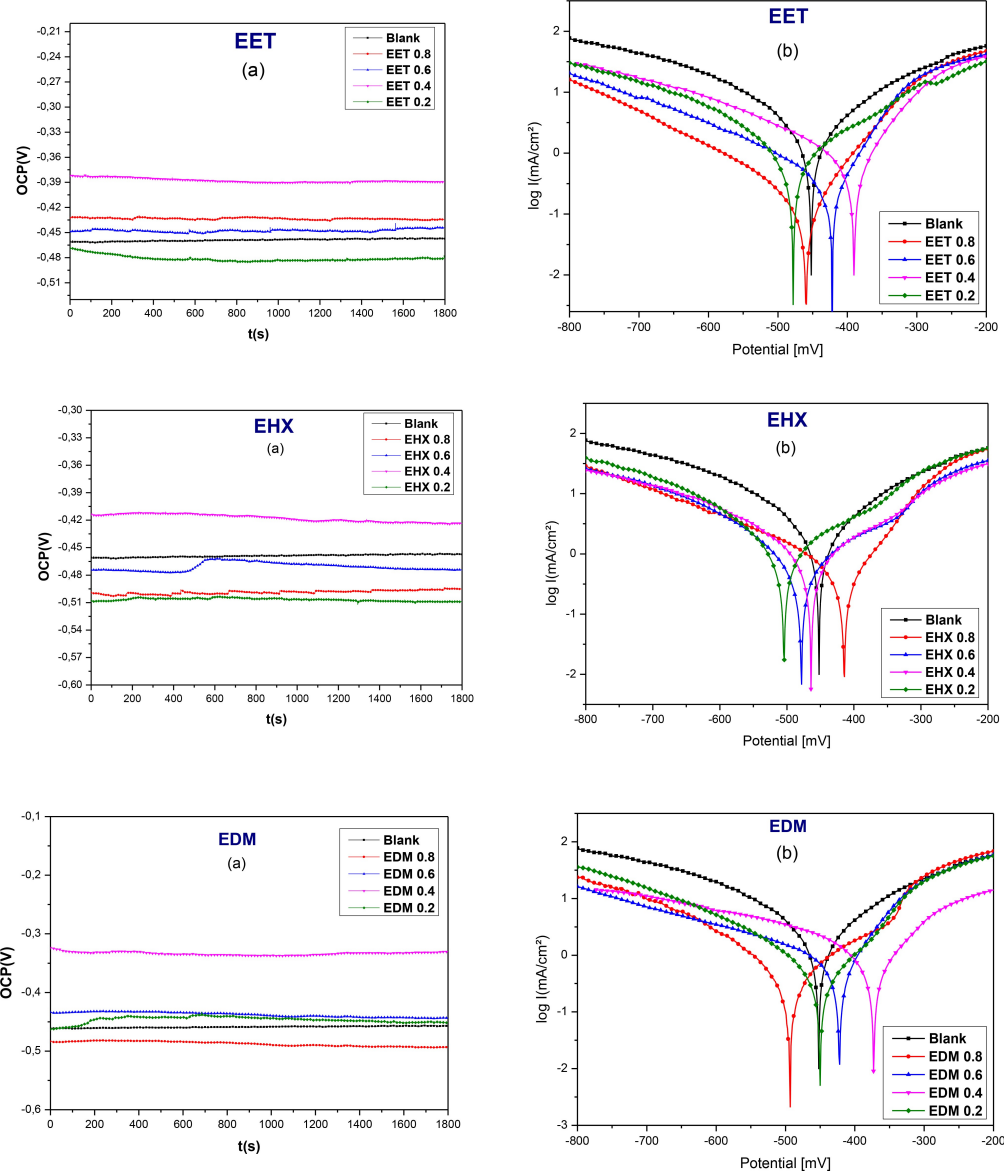
Evolution of the E_corr_ of steel as a function of time (a) and PDP curves at different concentrations of EET, EHX, and EDM (b).

Stabilizing the open‐circuit potential (OCP) is essential before conducting electrochemical experiments. Figure 7(a) presents the OCP measurement traces of the metal both with and without the addition of the inhibitor, as a function of time (s), at varying concentrations of EET, EHX, and EDM. Before reaching a steady state (after 30 minutes of immersion), the values of OCP were slightly increasing over time then became almost constant after 30 min, with and without the addition of the inhibitor of all concentrations, as an indication that open‐circuit potential reached equilibrium. When comparing OCP values with and without inhibitors, it is observed that the OCP values with various inhibitor concentrations are more positive than those of the blank, particularly with EET and EDM inhibitors. This positive shift is attributed to the adsorption of the inhibitor complexes on the steel surface, which blocks active anodic and cathodic corrosion sites.^[73]^ For EHX, adding its concentrations to a 1 M HCl solution shifts the OCP values to more negative potentials, indicating that it promotes oxide film dissolution. However, as the EHX concentration increases, this effect lessens due to its adsorption on the mild steel surface. The OCP shift can be explained by the formation of a protective inhibitor layer on the metal, which partially prevents corrosion reactions.^[74]^

Figure 7(b) illustrates the potentiodynamic polarization (PDP) plots for EET, EHX, and EDM inhibitors on mild steel in a 1 M HCl solution at a temperature of 308 K. These curves illustrate the behavior both in the presence and absence of different concentrations of inhibitors. Corrosion parameters including corrosion current density (*i_corr_*), corrosion potential (*E_corr_*), inhibition efficiency (*IE_PDP_*), as well as anodic and cathodic Tafel slopes (*β_c_* and *β_a_*) were extracted using Ec‐lab software and are documented in Table 5. The PDP plots in Figure 7 display straight lines parallel to the cathodic side for the three components extracted from C. *sativa L*, demonstrating that Tafel's law is followed and that the cathodic reaction is governed by a pure activation mechanism.^[58]^ This indicates that the addition of EET, EHX, and EDM to the corrosive medium does not influence the hydrogen reduction process, but instead, they effectively block the metal surface. In the Tafel plots, the current density is reduced for both the anodic and cathodic branches, signifying that the addition of EET, EHX, and EDM as an inhibitor reduces the current densities and causes a slight shift in the polarization curves toward the anodic side. These results confirm that the three major components extracted from C. *sativa L* function by blocking electrochemically active sites on the mild steel surface and forming a protective film.^[58]^

**Table 5:** Electrochemical parameters extracted from PDP curves vary with different concentrations of EET, EHX, and EDM inhibitors.

Inh	*Conc*. (g.L^−1^)	*I_corr_* (mA/cm^2^)	β_a_ (mV)	β_c_ (mV)	*E_corr_* (mV/SCE)	*IE_PDP_* (%)
	Blank	2.9973	172.5	−181.4	−452.2	
EET	0.2	1.5542	147.6	−232.5	−478.0	48
	0.4	0.9656	90.4	−224.3	−390.3	68
	0.6	0.4789	74.8	−228.2	−422.0	83
	0.8	0.2959	92.7	−195.9	−459.5	**90**
EHX	0.2	1.7140	184.0	−176.2	−504.4	43
	0.4	1.1447	176.0	−195.8	−463.9	62
	0.6	0.8778	160.7	−168.4	−478.4	71
	0.8	0.5242	84.1	−196.4	−414.6	**83**
EDM	0.2	2.0631	151.7	−279.6	−450.1	31
	0.4	1.2338	140.1	−283.2	−327.7	58
	0.6	0.9408	82.0	−315.2	−422.4	68
	0.8	0.5127	170.6	−144.5	−493.4	**82**

From the data in Table 5, it is evident that reduction in corrosion current density is notably more significant in the presence of the EET inhibitor compared to EHX and EDM, leading to an increase in inhibition efficiency. At a concentration of 0.8 g/L, EET exhibited its most effective inhibitory performance, reaching 90 % efficiency, with the corrosion current density decreasing to 0.2959 mA/cm^2^. This indicates that the inhibitor molecules initially adsorb onto the mild steel surface, blocking the available reaction sites and therefore reducing the corrosion current density. This observation is consistent with the results obtained from gravimetric measurements. Furthermore, altering the concentration of inhibitors has minimal influence on the displacement of *E_corr_*. The inhibitor's cathodic effect is more pronounced at both high and low concentrations of EET, low concentrations of EHX, and high concentrations of EDM. At intermediate concentrations of EET, high concentrations of EHX, and low concentrations of EDM, both anodic and cathodic current densities decrease, and the corrosion potential (*E_corr_*) shifts toward more anodic values. This indicates that the three main components extracted from C. *sativa L* act as mixed‐type corrosion inhibitors, with a predominant anodic effect. The loss of inhibition after a certain potential is likely due to the deformation or removal of the protective film from the metal surface.^[75]^ Table 5 shows significant changes in the anodic and cathodic Tafel slopes in the presence of inhibitors, though without a clear pattern. Increasing the concentration of the extracts in HCl solution causes slight alterations in the *βc* values. The cathodic current‐potential curves, both with and without the inhibitor, are nearly parallel, suggesting that the extracts from C. *sativa L* slow down the hydrogen evolution reaction without changing its mechanism. The protective surface film formed by the extracts reduces the reaction rate by preventing H^+^ reduction in the cathodic zones.^[76]^ In this case, corrosion occurs only in the exposed areas of the metal or through holes in the surface film. The best surface coverage was observed at 0.8 g/L for EET and EDM, where the film effectively closes the pores and acts as a better barrier between the metal and the corrosive environment. Conversely, the anodic process shows different behavior when the extracts are added. They slow down the metal dissolution reaction, reduce the anodic current density, and lower the *β_a_* values. This suggests that the extract molecules inhibit metal dissolution by altering the anodic reaction mechanism.[^77^, ^78^] However, further research is needed to fully understand this reaction mechanism. The results from potentiodynamic polarization (PDP) measurements align with those obtained from electrochemical impedance spectroscopy (EIS) and weight loss (WL) methods. The inhibition efficiency values can be categorized as follows: EET (90 %)>EHX (83 %)>EDM (82 %).

The inhibition efficiency of C*. sativa L*. ethanol extract (EET), reaching 90 %, can be compared to the corrosion inhibition potential of *Tamarindus indica* extract, which also contains naringenin as one of its components. In a study exploring the corrosion inhibition of mild steel (MS) in 1 M HCl, *Tamarindus indica* extract showed a high inhibition efficiency of 93 % at 800 ppm after 2.5 hours of immersion, as demonstrated by techniques such as electrochemical impedance spectroscopy (EIS), potentiodynamic polarization, scanning electron microscopy (SEM), and atomic force microscopy (AFM). Both extracts are highly effective, with *Tamarindus indica* slightly outperforming EET due to possibly more comprehensive physical adsorption on the steel surface.^[79]^ The dichloromethane extract of C. *sativa* L. (EDM), with 82 % inhibition efficiency, can be compared to Indonesian green tea extracts (GT1 and GT2), rich in catechins, which showed 75 % and 72 % inhibition in 1 M HCl, respectively. While these tea extracts performed well, EDM's stronger adsorption or more stable protective layers make it slightly more efficient.^[80]^ Similarly, the hexane extract of C. *sativa* L. (EHX), with 83 % efficiency, was compared to prickly pear seed oil extract (PPSO), which contains linoleic acid and demonstrated a maximum efficiency of 90 % in 1 M HCl. Variations in inhibition efficiency between these extracts may be attributed to differences in chemical composition and adsorption behavior.^[81]^ Other plant‐based inhibitors reported in the literature, such as *Ipomea staphylina* (92.77 %),^[82]^ *Luffa cylindrica* (87.89 %),^[83]^ *Petroselium sativum* (92.39 %),^[84]^ *Artemisia herba alba* (91 %),^[85]^ *Eichhornia crassipes* (86.8 %),^[86]^ *Phragmites australis* (89.6 %), and *Ocimum basilicum* (95.12 %),^[87]^ show similar efficiencies, emphasizing the strong corrosion inhibition potential of natural extracts. These comparisons highlight the effectiveness of C. *sativa* L. extracts, which perform on par with, or even exceed, other plant extracts in forming stable protective layers on steel surfaces.

### 2.3 Adsorption Isotherm

Adsorption isotherms offer crucial insights into the mechanism of organic molecule adsorption on a metal surface. Identifying the type of isotherm followed by the tested corrosion inhibitor is a fundamental step in this process. In our study, different kinds of adsorption isotherms including Langmuir, Freundlich, and Frumkin isotherms were studied based on the results obtained by the weight loss method in a 1 M HCl solution at a temperature of 308 K, and using their respective mathematical equations (Eqs. (10)–(12)[^88^, ^89^] to determine the most appropriate mode.

(11) $$Langmuir\;isotherm:\;\frac{C_{inh}}{\theta }=\frac{1}{K_{ads}}+C_{inh}$$

(12) $$Freundlich\;isotherm:\;ln\theta =ln\left(K_{ads}\right)+zln\left(C_{inh}\right)$$

(13) $$Frumkin\;isotherm:\;ln\left(\frac{\theta }{C_{inh}\left(1-\theta \right)}\right)=ln\left(K_{ads}\right)+sd\theta$$

**Figure 8:**
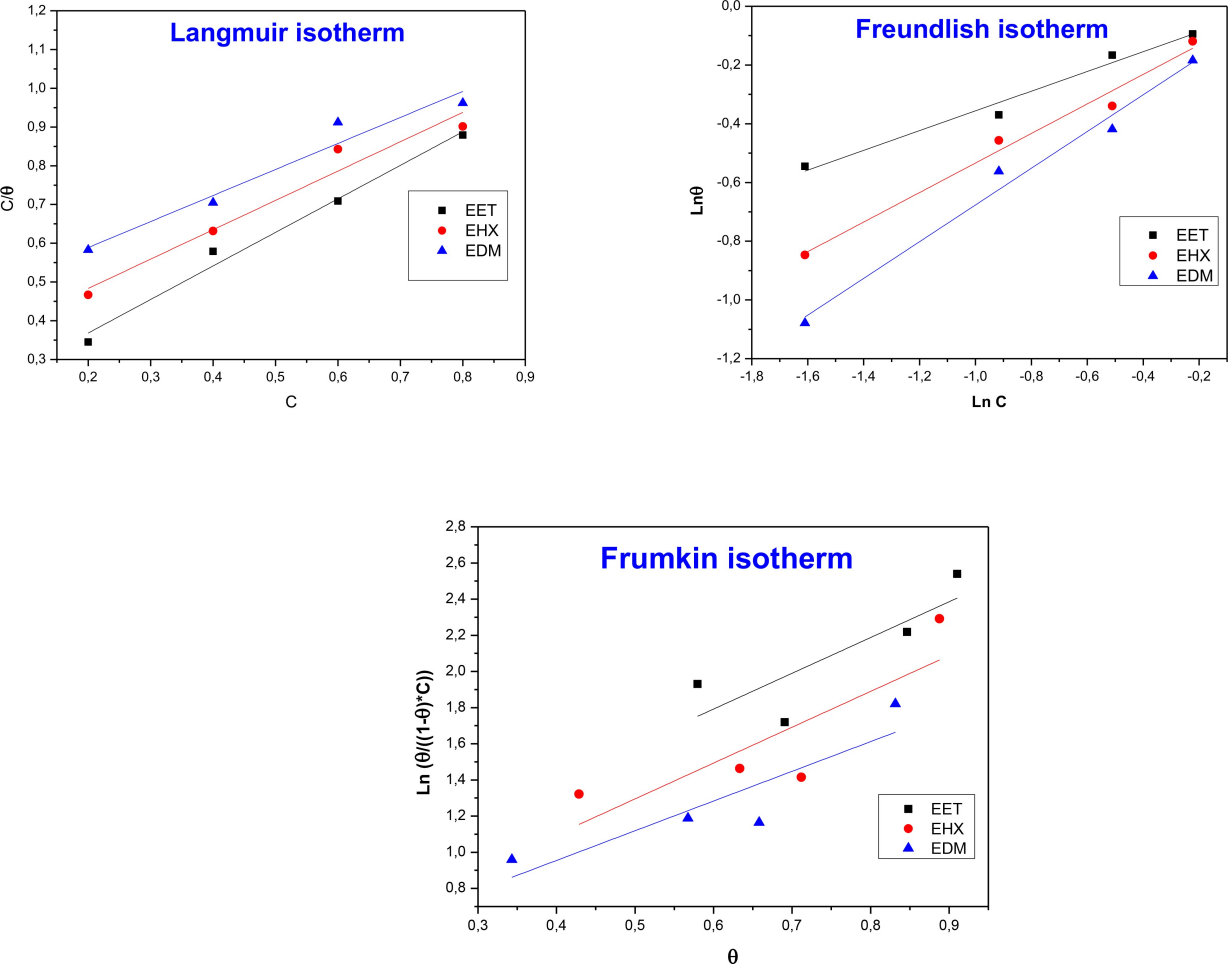
Langmuir, Freundlich and Frumkin adsorption isotherms for EET, EHX and EDM.

Figure 8 illustrates the adsorption isotherms obtained for EET, EHX, and EDM on the MS surface at 308 K. The values of linear correlation and slope from Table 6 for both Langmuir and Freundlich isotherms are close to unity, suggesting that the data fit well with both adsorption models. However, it is often useful to compare other factors such as the equilibrium constant *K_ads_* of adsorption derived from the slope and intercept of the lines of each model to determine the most appropriate model for describing the specific adsorption process. The values of *K_ads_* obtained from the Langmuir isotherm are higher compared to the other isotherms. This suggests that the adsorption of EET, EHX, and EDM on the metal surface is described by this isotherm. Langmuir's theory proposes the formation of a monolayer on a surface, suggesting that only one molecule of EET, EHX, or EDM per Fe adsorption site can be adsorbed and that the intermolecular forces decrease with distance. Additionally, it assumes that the metal surface displays homogeneous characteristics and possesses adsorption sites that are identical and energetically equivalent.^[90]^ The elevated values of the adsorption constants (*K_ads_*) indicate the substantial adsorption capacity of the tested EET, EHX and EDM on the surface of MS. Several authors agree that in the presence of natural extracts, it's safe to avoid evaluating the free enthalpy, 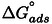 , because of the numerous compounds at different content cooperate to ensure corrosion protection of the metal. In other words, the inhibition process is interpreted by the synergistic intermolecular effect^[91]^ occurring via the biomolecules containing aromatic rings, double and triple bonds as well as heteroatoms (S, N, O …) as described by Figure 1. The nature of molecules like naringenin and Rutin, may also introduce the synergistic intramolecular effect.

**Table 6:** The values of *K_ads_* for various adsorption isotherms were tested.

Isotherm	Inh.	*R* ^*2*^	*K_ads_* (M^−1^)
Langmuir	EET	0.97209	3.0143
	EHX	0.97949	5.1245
	EDM	0.97742	2.2009
Freundlich	EET	0.96821	0.9801
	EHX	0.97547	0.9698
	EDM	0.97687	0.9498
Frumkin	EET	0.53805	1.8313
	EHX	0.55596	1.3557
	EDM	0.70594	1.3467

### 2.4 Theoretical Study

#### 2.4.1 Density Functional Theory Results

**Figure 9:**
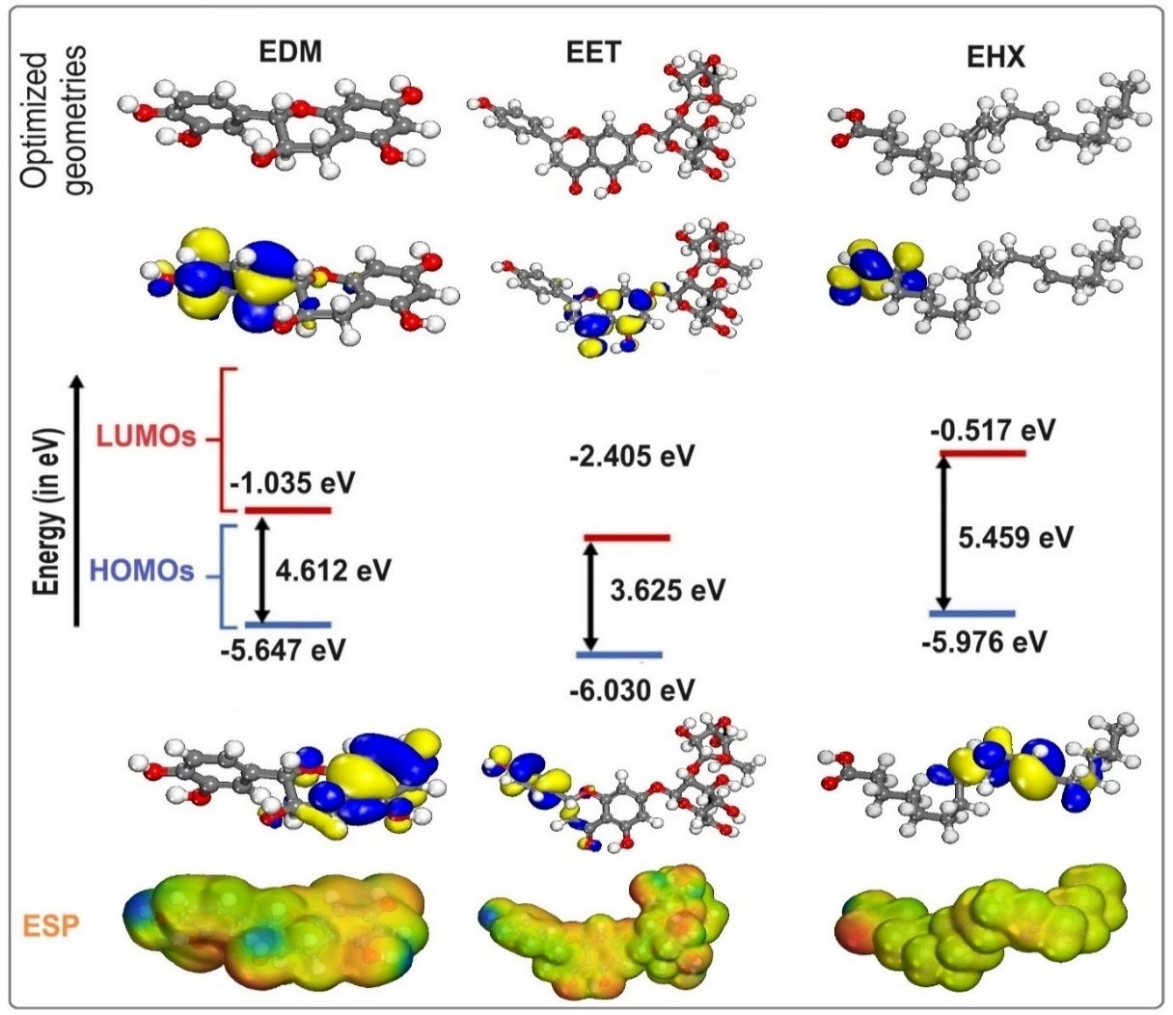
The optimized structures, as well as the HOMO, LUMO, and ESP images, are provided for EET, EHX, and EDM.

Theoretical investigations provide useful insight into the chemical activity of inhibitory compounds by applying electronic descriptors that define the features of these species and, consequently, the corrosion inhibition mechanisms.[^92^, ^93^, ^94^, ^95^, ^96^] The present study used three major constituents present in the investigated essential oil, namely EET, EHX, and EDM, to verify the structural effect on the experimentally acquired inhibition efficiency. The highest *E_HOMO_* value implies an increase in electron donor, implying improved corrosion inhibition efficiency due to higher adsorption of inhibitory compounds onto mild surfaces. Furthermore, the minimum *E_LUMO_* value denotes the capacity to admit electrons from the metal surface.^[97]^ Furthermore, a lower value of the gap energy *ΔE_gap_* represents the maximum interaction of the metal/inhibitor and, as a result, a high protection effectiveness. The optimized structures, as well as the FMOs (HOMOs/LUMOs) orbitals, and ESP images of EET, EHX, and EDM, are presented in Figure 9. According to the plots, the HOMO orbital for EDM and EET in the chosen molecules is spread across the entire molecule, focusing primarily on the aromatic doublets and hydroxyl groups. This result indicates that EDM and EET have a high electrophilic and nucleophilic nature. Furthermore, these FMOs are delocalized on the EHX moiety, as well as the −C=C− bonds and carboxyl, which are responsible for electron donation and/or acceptance at the metal surface. Furthermore, the red patches in the MEP represent the negative electrostatic potential, which has been enhanced around −C=C− bonds and oxygen atoms, as seen in Figure 9. Table 7 lists the quantum parameters for the EET, EHX, and EDM inhibitors. According to Table 7, the HOMO of the three compounds of C. *sativa L* essential oil is as follows: EDM>EHX>EET, indicating that EET has good electron‐donating properties to metal surfaces. According to Table 7, the theoretical order of ΔE and η is EET<EDM<EHX, implying that EET has excellent electron‐donating properties on the surface. Furthermore, the interaction energy will increase as the organic species’ rinsing softness increases. Furthermore, the ΔN and ΔEb‐d were computed (Table 7). It's notable that enhancing the electron‐donating capability of these inhibitors to donate electrons to the metal surface augments inhibition efficiency. As a result, the maximum fraction of electrons transmitted promotes the most efficient adsorption of the various components onto the steel surface, resulting in the best protection and inhibition. The *ΔEb‐d*<0 values for the three molecules indicate that back donation is preferable (Table 7). If *▵N*>0, *▵N* measures the transfer of electrons from molecule to metal; if *ΔN*<0, from metal to molecule.^[98]^ The positive figures obtained for the three compounds demonstrate their strong influence on steel corrosion.

**Table 7:** Electronic properties of EET, EHX, and EDM.

Compounds	*E_HOMO_* (eV)	*E_LUMO_* (eV)	*▵E* (eV)	*η* (eV)	*σ* (eV^−1^)	*χ* (eV)	*▵N* (eV)	*▵E*_*b–d*_ (eV)
EET	−6.030	−2.405	3.625	1.812	0.551	4.217	0.546	−0.453
EDM	−5.647	−1.035	4.612	2.306	0.433	3.341	0.320	−0.576
EHX	−5.976	−0.517	5.459	2.729	0.366	3.246	0.288	−0.682

#### 2.4.2 Monte Carlo and Molecular Dynamics Simulations

**Figure 10:**
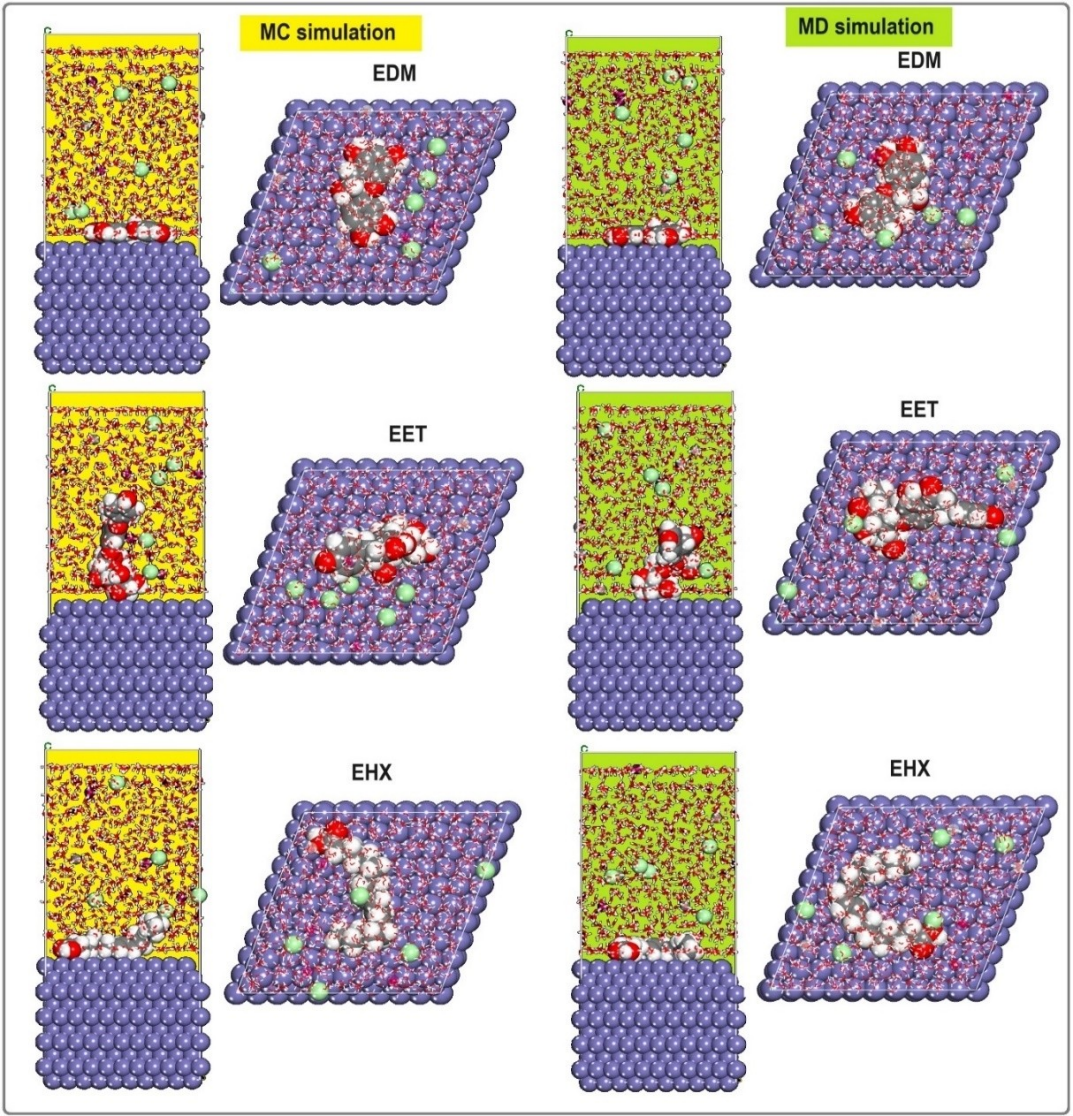
MC and MD results are provided for EET, EHX, and EDM.

**Figure 11:**
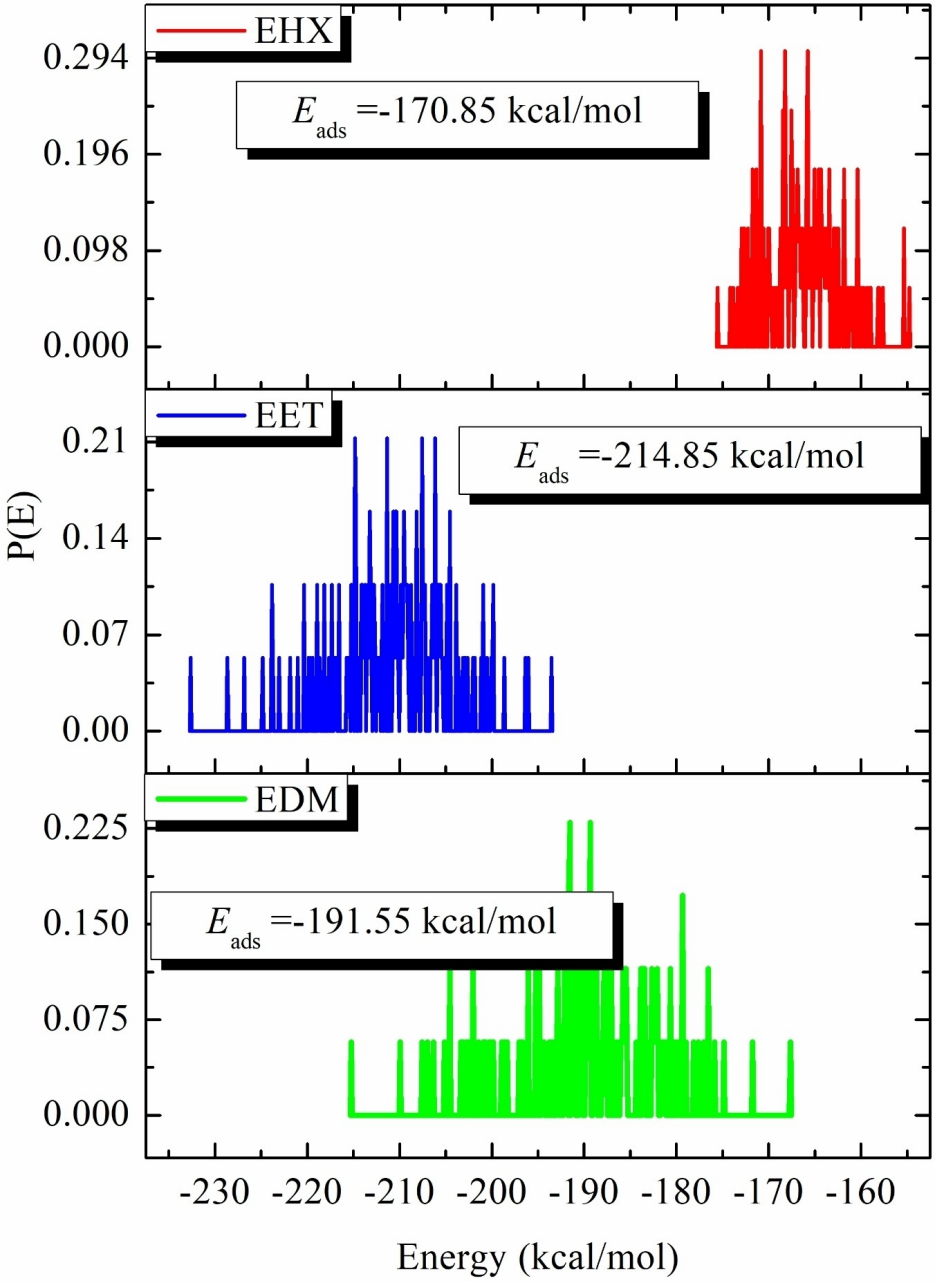
Distribution of the *E_ads_* of the EET, EHX, and EDM inhibitors on the Fe (110) surface via MC simulation.

**Figure 12:**
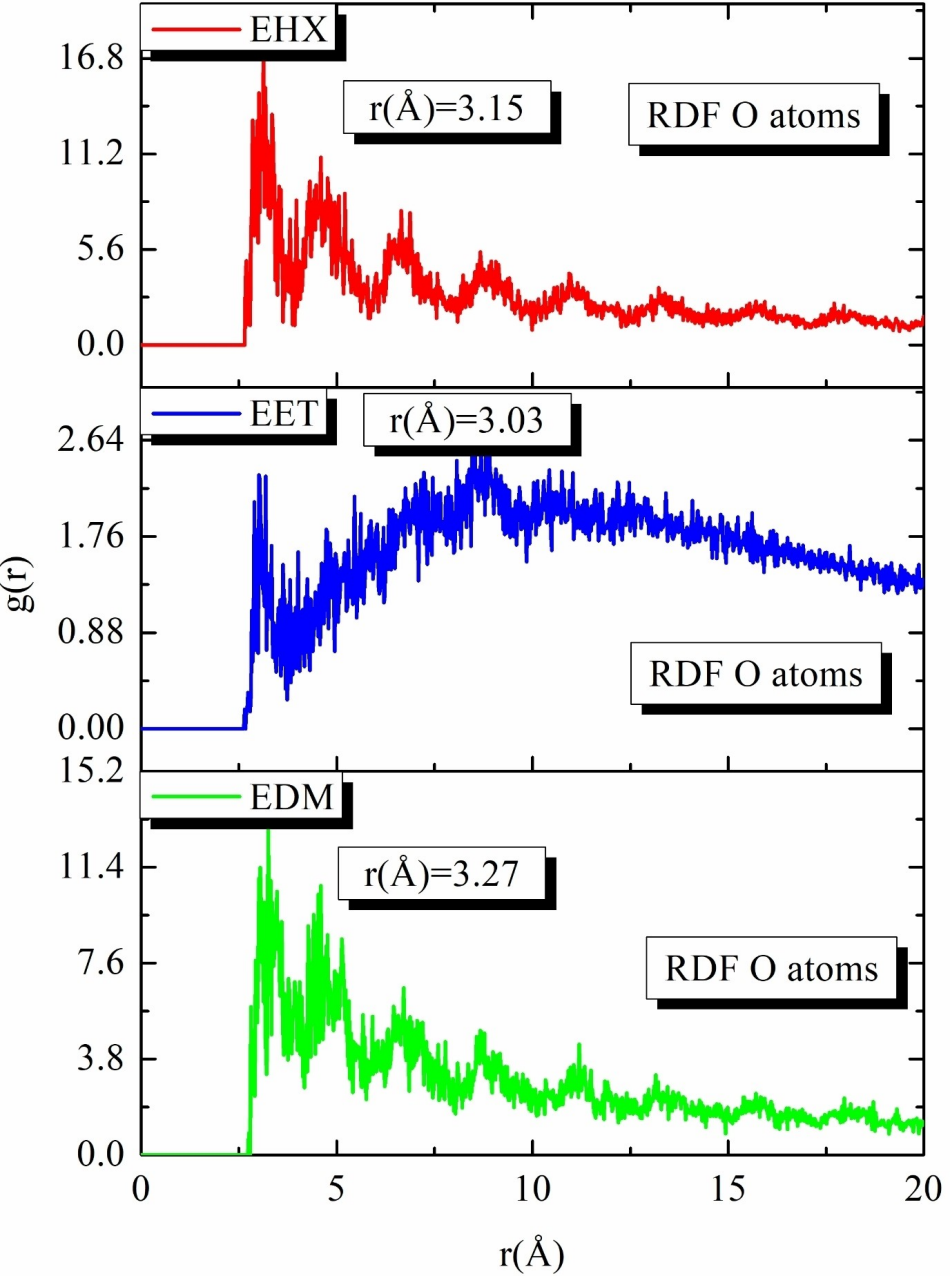
Temperature fluctuation (*T*=298 K) and RDF of the O atoms for EET, EHX, and EDM inhibitors, were obtained via MD.

Figure 10 presents this investigation of the use of Monte Carlo (MC) and Molecular Dynamics (MD) simulations to understand the adsorption behavior of EET, EHX, and EDM on an iron surface. This analysis is crucial for determining their efficacy as protective agents. The simulations revealed the low‐energy adsorption configurations of EET, EHX, and EDM on the Fe (110) surface. The three extracts of C. *sativa* L components exhibited a flat adsorption orientation, showing extensive surface coverage and effective protection. The presence of heteroatoms in EET, EHX and EDM such as oxygen, hydroxyl, carboxyl groups and −C=C− bonds, enhances their interaction with iron atoms. The EET, EHX and EDM molecules’ parallel orientation simplifies donor‐acceptor interactions, which enhances the protective coating on the metal surface. Figure 11 depicts the MC simulation distribution of Eads. According to these findings, the inhibitory potency of molecules is listed in the following order: EET (−214.85 kcal/mol)> EDM (−191.55 kcal/mol)> EHX (−170.85 kcal/mol). The compounds’ interactions on the Fe surface are stiffer due to the high binding energies. EET has demonstrated greater effectiveness compared to others due to its numerous active functional groups, primarily responsible for the heightened inhibitory efficiency. EET's O atom bonds come from higher adsorption of corrosion inhibitors. The RDF provides a probability density function for the distance between the inhibitor molecules and the iron atoms (Figure 12). Understanding the nature of interactions (whether physical or chemical bonds) between the inhibitor and the metal[^99^, ^100^] is crucial. The type of bonds formed at the inhibitor/metal interface plays a pivotal role in determining corrosion protection effectiveness. Bond length values were utilized to discern the various types of formed links. It is also revealed that the metal‐O bond values indicate the type of adsorptive activity that occurs over the metal. Chemisorption is thought to be present when the RDF peak is between 1 and 3.5, whereas bond length above this value exhibits physisorption behavior. The simulations show that EET, EHX, and EDM are very close to the metal surface, indicating a strong interaction. A robust interaction is necessary for the creation of a protective coating that effectively prevents corrosion. In summary, the molecular dynamics (MD) and Monte Carlo (MC) simulations played a crucial role in offering a detailed understanding of the adsorption behavior of estragol, linalol, and methyleugenol on iron surfaces. These insights are crucial for evaluating the potential of these materials as effective corrosion inhibitors in real‐world applications. A comprehensive analysis that combines experimental and computational approaches provides a reliable assessment of the inhibitory properties of these compounds.

## 3 Conclusions

The investigation into C. *sativa L* ethanol extract (EET), C. *sativa L* hexane extract (EHX), and C. *sativa L* dichloromethane extract (EDM) as potential green corrosion inhibitors for mild steel in an acidic medium (1 M HCl) have yielded several significant conclusions:

– The primary findings obtained through electrochemical techniques indicate that EET, EHX, and EDM function as effective and environmentally friendly corrosion inhibitors.
– The electrochemical impedance spectroscopy (EIS) results are consistent with the findings from weight loss measurements and potentiodynamic polarization (PDP) tests.
– PDP plots demonstrated the efficacy of EET, EHX, and EDM, with the corrosion current density reaching its minimum values at a concentration of 0.8 g/L for each inhibitor. This observation also indicates that EET, EHX, and EDM exhibit mixed‐type inhibition behavior.
– Thermodynamic analysis revealed that the inhibitors primarily act through physisorption adsorption modes.
– The investigation revealed that the adsorption process of EET, EHX, and EDM deposition on the metallic surface adhered to the Langmuir model.
– Monte Carlo (MC) and Molecular Dynamics (MD) simulations played an important role in providing a molecular‐level insight into the adsorption behavior of catechin dihydrate, naringenin and linoleic acid on iron surfaces. MC simulations also predicted high adsorption energies, consistent with our experimental findings.
– This study enhances our understanding of C. *sativa L*. as a source of bioactive compounds, opening new research opportunities for hemp products in pharmaceuticals, food, cosmetics, and other industries. The abundant availability of Cannabis in regions like Morocco presents exciting potential for large‐scale use. However, to effectively utilize Cannabis extracts as corrosion inhibitors, assessing their stability under real‐world conditions is essential to ensure their efficacy and longevity in industrial applications.

## Funding

This research is funded by Researchers Supporting Project number (RSP2024R132), King Saud University, Riyadh, Saudi Arabia.

## Author Contributions

Conceptualization, S.H., B.H and A.C.; methodology, S.H., K.Z., and A.H.; software, H.K., M.B., and M.A.M.; validation, M.B., A.A., B.H., and A.C.; formal analysis, M.B.; investigation, S.H., B.H., A.C.; resources, A.A.; data curation, S.H.; writing – original draft preparation, S.H., A.H., K.Z., O.D.; writing – review and editing, H.K., M.B., S.H., K.Z., A.H., O.D., A.S.A., O.M.N., A.A.; visualization, M.B., A.A., B.H., A.S.A., O.M.N., M.A.M.; supervision, A.C.; funding acquisition, A.S.A., O.M.N. All authors have read and agreed to the published version of the manuscript.

## Conflict of Interests

The authors declare no conflict of interest.

4

## Data Availability

The original contributions presented in the study are included in the article. Further inquiries can be directed to the corresponding author.
